# Supplementary figures and images for: Effects of larval foam-making and prolonged terrestriality on morphology, nitrogen excretion and development to metamorphosis in a Leptodactylid frog
Source: PeerJ. 2025 Feb 26;13:e18990. doi: 10.7717/peerj.18990 (PMC11871897; doi:10.7717/peerj.18990)

A) 14.5 d, 1 nest constructed

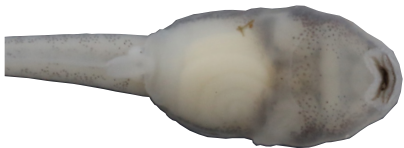

B) 18.5 d, 3 nests constructed

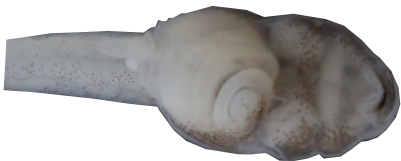

C) 14.5 d, 2 d in water, unfed

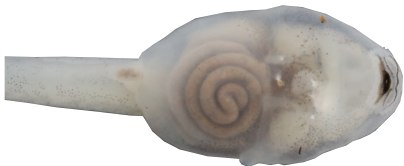

Supplement: Supplemental Information 1 — Larvae at (A) 14.5 days, after constructing a new larval foam nest, (B) 18.5 days, after constructing three larval nests. and (C) 2 days in aged, dechlorinated tap water with no food provided. [file peerj-13-18990-s001.pdf]
