# Supplementary material for: Effects of larval foam-making and prolonged terrestriality on morphology, nitrogen excretion and development to metamorphosis in a Leptodactylid frog
Source: PeerJ. 2025 Feb 26;13:e18990. doi: 10.7717/peerj.18990 (PMC11871897; doi:10.7717/peerj.18990)
Supplement: Supplemental Information 4 [file peerj-13-18990-s004.pdf]

Supplementary Tables

Table S1. Coefficient of variation (%CV) of seven morphometric variables of *Leptodactylus fragilis* larvae for five randomly selected sibships after 12.5 days in a parental foam nest in soil, plus either two days in water or after making one, two or three larval nests on land. We measured a total of 29 photographs three times for each morphometric variable to obtain CVs.

Measurement	Larval nest 1 (N=8)	Larval nest 2 (N=8)	Larval nest 3 (N=8)	Water (N=5)	Total (N=29)
Total Length (TL)	0.33%	0.26%	0.13%	0.21%	0.25%
Tail Length (TAL)	0.92%	0.81%	0.61%	0.55%	0.79%
Tail Muscle Width (TMW)	2.62%	0.62%	1.77%	1.65%	1.75%
Interorbital Distance (IOD)	2.97%	1.67%	3.68%	2.07%	2.78%
Head Width (HW)	0.59%	0.31%	0.60%	0.31%	0.49%
Tail Muscle Height (TMH)	1.69%	1.57%	2.05%	1.15%	1.76%
Tail Height (TH)	0.97%	1.18%	1.38%	0.95%	1.21%

Table S2. Total volume of foam (mm^3) produced by sibling groups of *Leptodactylus fragils* larvae removed from their parental nest on soil at three ages and stages relative to their entry into developmental arrest. Consecutive nests were constructed following removal of larval foam at 2-day intervals. Data are means ± SD and number of sibships (**N**).

	Age (stage of development) at loss of parental foam nest		
	4.5 d (pre-arrest)	8.5 d (at arrest)	12.5 d (after arrest)
First larval nest	5586 ± 1891, **14**	6539 ± 1375, **8**	6319 ± 1297, **22**
Second larval nest	6904 ± 1234, **6**	6180 ± 1827, **8**	6258 ± 1199, **15**
Third larval nest		6402 ± 2462, **5**	6577 ± 1258, **15**

Table S3. Eigenvalues and percentage of cumulative variance of the first five principal components (PC) from 7 common morphometric measurements of early larvae after extended development in their parental nest on land (to 12.5 days) and a variable period of development either making new larval foam nests on land (to 14.5, 16.5 and 18.5 days) or in water (to 14.5 days).

	**Foam vs water (2 days)**		**Larval foam: 1st—3rd nest**	
Principal Component	Eigenvalue	Cumulative % of variance	Eigenvalue	Cumulative % of variance
PC 1	4.85	69.25	5.80	65.97
PC 2	0.91	82.23	1.27	82.02
PC 3	0.67	91.84	0.80	88.64

Table S4. Percentage contribution (A) and coordinates (B) of seven morphometric variables for the first three principal components (PC) of larvae of *Leptodactylus fragilis* after extended development on land (12.5 days) and variable periods of development either making new larval foam nests (to 14.5, 16.5 and 18.5 days) or in water (14 days). Variable contributions above ten percent are in bold.

Measurement	**Foam vs water (2 days)**			**Larval foam: 1st—3rd nest**		
**A) Contribution**	PC 1	PC 2	PC 3	PC 1	PC 2	PC 3
Total Length (TL)	**19.66**	1.00	0.01	**18.15**	8.03	1.52
Tail Length (TAL)	**18.45**	2.93	0.94	**16.92**	**12.05**	1.56
Tail Muscle Width (TMW)	8.93	4.99	**73.39**	**15.11**	8.35	**27.68**
Interorbital distance (IOD)	**15.94**	1.28	0.00	**13.86**	9.26	**40.63**
Head Width (HW)	**16.79**	0.05	8.14	**18.20**	1.53	7.31
Tail Muscle Height (TMH)	3.23	**87.43**	6.15	3.88	**58.04**	**10.50**
Tail Height (TH)	**16.99**	2.29	11.34	**13.86**	2.74	**10.81**
**B) Coordinates**	PC 1	PC 2	PC 3	PC 1	PC 2	PC 3
Total Length (TL)	0.98	-0.09	0.01	0.91	-0.30	-0.08
Tail Length (TAL)	0.94	-0.16	0.08	0.88	-0.37	-0.08
Tail Muscle Width (TMW)	0.65	0.21	0.70	0.84	0.31	-0.36
Interorbital distance (IOD)	0.88	-0.10	0.00	0.80	-0.32	0.43
Head Width (HW)	0.90	0.02	-0.23	0.92	0.13	-0.18
Tail Muscle Height (TMH)	0.39	0.89	0.20	0.42	0.81	0.22
Tail Height (TH)	0.91	-0.14	-0.28	0.80	0.18	0.22

Table S5. Time to and size and mass at metamorphosis in *Leptodactylus fragilis* that entered the water after making zero, one, or three larval foam nests. Times (days) from oviposition (age) and water entry (aquatic period) to forelimb emergence and to tail resorption. Data are means ± SD and number of sibships (**N**).

**Forelimb emergence (Gosner stage 42)**				
**Larval nests**	**Age (days)**	**Aquatic period (days)**	**Total length (mm)**	**Mass (mg)**
**0**	30.27 ± 2.26, **30**	18.27 ± 2.26, **30**	38.04 ± 3.90, **29**	0.31 ± 0.10, **18**
**1**	31.78 ± 1.72, **40**	17.78 ± 1.72, **40**	37.70 ± 4.29, **38**	0.34 ± 0.10, **14**
**3**	36.72 ± 2.98**, 39**	18.72 ± 2.98, **39**	37.40 ± 3.40, **39**	0.30 ± 0.08, **18**
**Tail resorption (Gosner stage 46)**				
**Larval nests**	**Age (days)**	**Aquatic larval + metamorphic period (days)**	**SVL (mm)**	**Mass (mg)**
**0**	37.00 ± 2.39, **18**	25.00 ± 2.39, **24**	12.14 ± 1.21, **24**	0.21 ± 0.06, **23**
**1**	38.84 ± 2.84, **32**	24.84 ± 2.84, **32**	11.99 ± 1.01, **31**	0.20 ± 0.05, **31**
**3**	43.72 ± 3.28**, 18**	25.72 ± 3.28, **32**	11.55 ± 0.91, **29**	0.19 ± 0.05, **26**
